# Awareness of Obstetric Danger Signs Among Pregnant Women Attending a Tertiary Care Hospital in Hyderabad, Telangana: A Cross-Sectional Study

**DOI:** 10.7759/cureus.89132

**Published:** 2025-07-31

**Authors:** Garima Misra, Durga A, Sai Sadhgun Sangem, Vikas Saharan

**Affiliations:** 1 Department of Community Medicine, Employees' State Insurance Corporation (ESIC) Medical College and Hospital, Hyderabad, IND

**Keywords:** antenatal, obstetric danger signs, pregnancy complications, pregnant women, warning signs

## Abstract

Introduction

Knowledge of obstetric danger signs among pregnant women can facilitate the timely recognition of obstetric danger signs, thereby leading to a reduction in overall maternal morbidity and mortality. Our cross-sectional study assessed the level of awareness among pregnant women about these obstetric danger signs and understood the influence of sociodemographic factors and obstetric history on their knowledge.

Methods

The study enrolled 165 participants who were pregnant women attending the antenatal outpatient department of a tertiary care hospital in Telangana. The participants, who gave consent for the study, were followed over two months and were surveyed using a semi-structured, interviewer-based questionnaire. This not only evaluated their awareness about obstetric danger signs but also included questions on their sociodemographic and obstetric profile. Their knowledge was graded in terms of a score of 0-10 based on the questions asked. Data were further analyzed using Microsoft Excel (Microsoft Corp., Redmond, WA, US) and GraphPad Prism Software version 5 (Insight Venture Management, LLC, New York, NY, US).

Results

All the study participants (165) gave consent for the survey. Our study concluded that about 64.84% (107 out of 165) of the pregnant women had adequate knowledge about obstetric danger signs. In addition to this, there was a statistically significant (p < 0.05) association noted between adequate knowledge and occupation as a homemaker (53.37%, 57 out of 107), having their second pregnancy (36.44%, 39 out of 107), and a history of zero miscarriages (58.87%, 63 out of 107).

Conclusions

The study concluded that more than half of the participants had adequate knowledge, which could contribute significantly to improved maternal outcomes. The findings also highlight the various factors associated with awareness that can help tailor healthcare initiatives and policies in the long term.

## Introduction

The objective of the study was to assess the level of awareness about obstetric danger signs among pregnant women and to identify the factors, such as sociodemographic features and obstetric history, that influence their understanding of obstetric danger signs in Telangana, India. The World Health Organization has defined maternal mortality as “death of women during pregnancy or within forty-two days of termination of pregnancy, irrespective of duration and site of pregnancy, from any cause related to or as a complication of pregnancy or its management, but is not attributed to accidental or incidental causes.” Delays that cause most maternal mortalities, also called the 3 D’s, are a delay in the decision to seek care, a delay in reaching the place of care, and a delay in getting appropriate and sufficient care [1]. To decrease preventable maternal mortality, providing health education to all parties involved in healthcare, including patients, families, communities, and medical personnel, is mandatory. Good knowledge will lead to appropriate attitudes and practices, i.e., prompt referral to proper medical services, thus decreasing preventable maternal mortality. Unawareness of obstetric danger signs will delay the decision to seek proper care and eventually lead to morbidity and mortality. The commonest danger signs during pregnancy include severe vaginal bleeding, swollen hands/face, and blurred vision. Raising awareness among pregnant women of the danger signs would improve the early detection of problems and reduce the delay in deciding to seek obstetric care. Thus, one of the key strategies for reducing maternal mortality is increasing knowledge of the obstetric danger signs among women, family, and the community at large [2]. The mother and family should immediately seek medical help if obstetric danger signs are present. There is significant variation in the statistics of mothers seeking help for obstetric danger signs by utilizing antenatal care (ANC) visits, a minimum of four visits during the pregnancy period, as compared to the women who did not seek help. Some developing countries reported their statistics for seeking help as follows: the number of women who sought prenatal help in India, Tanzania, Indonesia, and Ethiopia was 72%, 64.7%, 96.9%, and 29.1%, respectively. This variation of statistics highlights the need to improve maternal education effectiveness and healthcare access in developing countries [3]. Combining education with other efforts to reduce maternal morbidity and mortality, such as better access to healthcare facilities, could help the United Nations meet its Sustainable Development Goal 3.1 of reducing maternal mortality to below 70 per 100,000 live births by 2030. Similarly, our study elucidates the present knowledge among pregnant women regarding obstetric danger signs that, in turn, would have a positive impact in reducing maternal morbidity and mortality [4].

## Materials and methods

This cross-sectional study was conducted at the antenatal outpatient department (OPD) of Employees’ State Insurance Corporation (ESIC) Medical College, Hyderabad. The study was carried out over two months, specifically from February 1, 2025, to April 1, 2025. The research aimed to assess the knowledge of obstetric danger signs among pregnant women attending routine antenatal check-ups at the institution. The Institutional Ethics Committee has reviewed the project bearing no. ESICMC/SNR/IEC-S0490/01-2025, version no. V01, and unanimously approved the proposed project.

The study population consisted of all pregnant women attending the antenatal OPD during the study period. Women were included in the study if they were pregnant, attending the antenatal clinic at ESIC Medical College, and provided written informed consent to participate (inclusion criteria). Those who were not pregnant, declined to participate, or were found to be in a critical condition requiring emergency obstetric care at the time of recruitment were excluded to ensure patient safety and focus on the educational aspects of the study (exclusion criteria).

To determine the appropriate sample size, the researchers referred to a prior study conducted in Maharashtra, India, by Vijay et al. [5], which evaluated the knowledge of obstetric danger signs among the study participants. Based on that study, the proportion of women who were aware of obstetric danger signs (p) was estimated at 0.27. The complement of this proportion (q) was 0.73, and a margin of error (e) of 0.07 was used. Using the standard formula for sample size calculation in cross-sectional studies,

$n=\frac{Z^{2}&sdot;p&sdot;q}{e^{2}}$​​​​​​​

$n=\frac{(1.96 &times; 1.96 &times; 0.27 &times; 0.73)}{0.07 &times; 0.07} = 154$

where Z represents the standard normal variate for a 95% confidence interval (CI) (1.96), the minimum sample size was calculated as 154. However, to account for non-responses and possible exclusions during data cleaning, a total of 165 pregnant women were ultimately enrolled in the study.

Participants were recruited consecutively as they presented to the antenatal OPD, and data collection continued until the desired sample size was reached. A semi-structured, interviewer-administered questionnaire (referred to in the appendix) was used to gather data. To ensure comprehensibility and improve the validity and reliability of the responses, the questionnaire was translated from English into Telugu and Hindi, depending on the participant’s preferred language. Interviews were conducted in a private setting to ensure confidentiality and to promote open and honest communication.

The self-adapted questionnaire designed for this study had two sections. The first section focused on the sociodemographic characteristics of the participants, including their age, occupation, and place of residence. It also collected relevant obstetric history such as the total number of pregnancies, previous abortions or any miscarriages, and age at first pregnancy. The second section was specifically designed to assess the participant’s knowledge of 10 critical obstetric danger signs. These included severe headache, blurred vision, elevated blood pressure, leaking per vagina without labor pains, facial swelling and generalized edema, burning micturition or abnormal vaginal discharge, vaginal bleeding, fever, severe abdominal pain, and decreased fetal movements. Each correctly identified danger sign was awarded one point, resulting in a total possible score of 10. Participants scoring five or more were considered to have adequate knowledge, whereas those scoring below five were categorized as having poor knowledge of obstetric danger signs.

All collected data were entered into Microsoft Excel (Microsoft Corp., Redmond, WA, US) and analyzed using GraphPad Prism software version 5 (Insight Venture Management, LLC, New York, NY, US). Descriptive statistics such as mean and standard deviation were used for continuous variables, while frequencies and percentages summarized categorical variables. The associations between different sociodemographic and obstetric variables and the level of knowledge were tested using the Chi-squared test or Fisher’s exact test, depending on the distribution and sample sizes. A p-value less than 0.05 was considered statistically significant.

Ethical clearance for the study was obtained from the Institutional Ethics Committee of ESIC Medical College, Hyderabad, prior to commencement. Written informed consent was obtained from all participants after explaining the purpose, procedures, and their rights, including the right to withdraw at any point without affecting their medical care.

## Results

A total of 165 pregnant women who fulfilled the inclusion criteria were included in this study. The mean age of the study participants was 26.3 ± 4.66 years. The sociodemographic and obstetric profiles of the women are presented in Tables 1, 2, respectively.

**Table 1:** Distribution of the sociodemographic characteristics of the study participants The statistical test used for the calculation of the p-values was the Chi-squared test. A p-value less than 0.05 was considered to be statistically significant

Socioeconomic features	Knowledge		N (total = 165)	Percentage	p-value
	Adequate	Poor			
Present age					0.0991
<19 years	4	9	13	7.87%	
20-25	43	23	66	40.00%	
26-30	39	17	56	33.93%	
31-35	18	7	25	15.15%	
>36 years	3	2	5	3.05%	
Total	107	58	165	100%	
Residence					0.7128
Urban	77	44	121	73.33%	
Rural	30	14	44	26.67%	
Total	107	58	165	100%	
Educational qualifications					0.4937
Illiterate	13	7	20	12.12%	
5th-10th grade	30	17	47	28.48%	
12th grade	22	18	40	24.24%	
Degree	28	13	41	24.85%	
Postgraduate	7	2	9	5.46%	
Up to 10th grade	7	1	8	4.85%	
Total	107	58	165	100%	
Occupation					0.0199
Housewife	57	42	99	60.00%	
Working individual	50	16	66	40.00%	
Total	107	58	165	100%	

**Table 2:** Distribution of the obstetric characteristics of the study participants The statistical test used for the calculation of the p-values was the Chi-squared test. A p-value less than 0.05 was considered to be statistically significant

Obstetric history	Knowledge		N (total = 165)	Percentage	p-value
Variables	Adequate	Poor			
Pregnancies					0.0084
1	23	23	46	27.88%	
2	39	24	63	38.18%	
3	24	5	29	17.58%	
4	12	6	18	10.91%	
5	7	0	7	4.24%	
6	2	0	2	1.21%	
Total	107	58	165	100%	
Number of miscarriages					0.0387
0	63	43	106	64.24%	
1	31	14	45	27.27%	
2	12	0	12	7.27%	
3	0	1	1	0.61%	
4	1	0	1	0.61%	
Total	107	58	165	100%	
Age during first pregnancy					0.1224
<19 years	17	18	35	21.21%	
20-25 years	71	29	100	60.61%	
26-30 years	16	9	25	15.15%	
31-35 years	3	2	5	3.03%	
Total	107	58	165	100%	

The majority of the participants belonged to the age group of 20-25 years (66, 40.00%), and the largest proportion of participants in the study resided in urban areas (121, 73.33%). Most of the women were educated up to the level of 5th-10th grade (47, 28.48%). The greatest proportion of pregnant women who participated in the study were homemakers (99, 60.00%). The study participants’ obstetric history showed that the majority had two pregnancies (63, 38.18%) and zero miscarriages (106, 64.24%). The maximum number of women had their first pregnancy in the age group of 20-25 years (100, 60.61%).

On analysis, it was found that 64.84% (107 out of 165) of the study participants had adequate knowledge (score of 5 and above), and 35.15% (58 out of 165) had poor knowledge (score below 5) about obstetric danger signs during pregnancy. The proportion of the study participants having adequate and poor knowledge is depicted in Table 3.

**Table 3:** Proportion of the study participants having adequate and poor knowledge Adequate knowledge: score of 5 and above; poor knowledge: score below 5

Knowledge regarding obstetric danger signs among the study subjects	Number (n)	Percentage
Adequate knowledge	107	64.84
Poor knowledge	58	35.15

The most recognized obstetric danger signs among study participants were severe abdominal pain (132, 80.00%), followed closely by bleeding per vagina (130, 78.79%). In contrast, the participants had the least awareness about swelling of hands and face (43, 26.06%) as a danger sign. The study participants' knowledge of obstetric danger signs during pregnancy is presented in Table 4.

**Table 4:** Distribution of the knowledge of obstetric danger signs during pregnancy in the study participants

Obstetric danger sign	Awareness	N (total = 165)	Percentage
Severe abdominal pain	Aware	132	80.00%
				Not aware	33	20.00%
	Blurred vision	Aware	59	35.76%
Not aware					106	64.24%
Elevated blood pressure		Aware	114	69.09%
	Not aware				51	30.91%
	Decreased fetal movements	Aware	107	64.85%
Not aware					58	35.15%
Bleeding per vagina		Aware	130	78.79%
	Not aware				35	21.21%
	Leaking per vagina without labor	Aware	118	71.52%
Not aware					47	28.48%
Burning micturition/foul-smelling vaginal discharge		Aware	45	27.27%
	Not aware				120	72.73%
	Swelling of hands and face	Aware	43	26.06%
Not aware					122	73.94%
Fever		Aware	62	37.58%
	Not aware				103	62.42%
	Severe headache	Aware	57	34.55%
Not aware					108	65.45%

The study demonstrated that among all the pregnant women who had adequate knowledge, the highest and lowest numbers belonged to the age group of 25-30 years (43 out of 107, 40.18%) and <19 years (four out of 107, 3.73%), but this was not found to be statistically significant. The interpretation of the determining factors and their association with knowledge among pregnant women reveals that 71.96% of the women who had adequate knowledge resided in urban areas. However, this was not found to be statistically significant, probably because the survey was conducted in an urban tertiary care center. Among each category of educational qualifications, all the categories had a greater number of people with adequate knowledge than those with poor knowledge. Although statistically insignificant, women with an education of 5th-10th grade (28.03%, 30 out of 107) made the largest proportion of study participants having adequate knowledge, followed by individuals who had a degree (26.16%, 28 out of 107). Out of all the women who had adequate knowledge in the study, the greatest proportion were homemakers (53.27%, 57 out of 107), and this association between occupation and level of knowledge was found to be statistically significant (p = 0.0199). Another statistically significant (p = 0.0084) correlation was found between the number of pregnancies and the level of knowledge, in which out of all the study participants who had adequate knowledge, the majority had two pregnancies (36.44%, 39 out of 107). The study results also revealed that the women having zero miscarriages constituted the highest proportion among the participants who had adequate knowledge (58.87%, 63 out of 107). Although the latter association was statistically significant (p = 0.0387), this could be attributed to the smaller study sample, which coincidentally had a higher proportion of pregnant women with no prior abortions.

## Discussion

Our study assessed the awareness of obstetric danger signs among pregnant women in a tertiary care hospital and scored their responses on a scale of 0-10. It was found that more than half (64.84%) of the participants in the study had adequate knowledge (score of 5 and above). Likewise, in a study conducted by Haleema et al. in Karnataka, with a similar sample size and scoring pattern, it was found that 54.7% of the participants had adequate knowledge [6]. Contrastingly, another study conducted by Imani Ramazani et al. [7], in the Eastern Democratic Republic of Congo, revealed a lower proportion (21.9%) of knowledge about the obstetric danger signs. After conducting a survey and analyzing the data for the present study, it was concluded that the highest number of pregnant women had awareness about severe abdominal pain (80%), followed by bleeding per vagina as one of the obstetric danger signs (78.79%). However, in a study done by Alshaikh et al. in Abha city, bleeding per vagina (dropping) (73.8%) was reported as the most common obstetric danger sign that the pregnant women were aware of [8]. This research also highlighted the correlation between the sociodemographic and obstetric profiles of the participants and their level of knowledge. In the present study, most of the women having adequate knowledge belonged to the age group of 25-30 years (40.18%). This resembled a study conducted by Hibstu and Siyoum [9] in Southern Ethiopia, where it was found that women in the age group 25-34 years were 3.5 (adjusted odds ratio (AOR) = 3.68, 95% CI: 1.30-10.46) times more likely to be knowledgeable of obstetric danger signs in relation to women who were 35 years old and above. Our study also concluded that the majority of the participants having adequate knowledge resided in urban areas; however, this was not statistically significant. These results were quite similar to a study conducted in Rwanda by Kayiganwa and Okova [10], which showed that participants residing in urban areas were three times more likely to be associated with a high level of knowledge on obstetric danger signs, with AOR = 3.812, 95% CI: 2.283-6.366, p < 0.001. On analysis, a statistically significant correlation was found between adequate knowledge and employment, where the homemakers had a better understanding of obstetric danger signs as compared to the employed women. This could be due to the small study sample and a higher proportion of homemakers among the study participants, who outnumbered the employed women. These findings were contrastingly different from a study conducted by Bolanko et al. [11] in Southern Ethiopia, which asserted a statistically significant association between employment and good knowledge of obstetric danger signs. Due to the higher proportion of participants in our study having education up to the secondary level, these participants constituted the largest number of women having adequate knowledge. However, this was statistically insignificant and probably did not reflect the true distribution in the population. Other studies highlighted a positive and significant association between women's knowledge of obstetric danger sign and their level of education. The latter results were depicted in a study conducted by Asfaha et al. in the southeast zone of Tigray, in which women who had a diploma and above level of education were AOR = 2.7, 95% CI: 1.189-6.24 more likely to have good knowledge on obstetric danger signs as compared to those reproductive-age women who were not educated/illiterate [12]. A study done by Nkamba et al. in the Democratic Republic of Congo revealed that the number of obstetric danger signs mentioned by the women was significantly higher in multigravida women than in primigravida women (Adj. incidence rate ratio (IRR) = 1.38; 95% CI: 1.23-1.55) [13]. These findings resembled the statistically significant association found in our study, where the multigravida women were more aware of the danger signs as compared to the primigravida women. Our study also exemplified the significant association between the number of prior miscarriages and the awareness of pregnant women regarding danger signs, where women who had zero miscarriages had the highest level of awareness. This correlation was determined in very few studies.

One of the limitations of the study could be attributed to the small sample size. Moreover, the study was conducted in a tertiary care hospital, and it may suffer from non-representative sampling, limiting its generalizability. Qualitative methods need to be supplemented with KAP (knowledge, attitude, and practice) studies for a more comprehensive understanding. Behavior change is influenced not only by knowledge but also by factors such as social norms, access to healthcare, and a supportive environment. Recall bias may also play a role, as participants may inaccurately remember or report past behaviors or events. Future studies could build upon these findings by incorporating multivariate analysis and exploring theoretical frameworks that would provide a more comprehensive understanding of the topic.

## Conclusions

Improved awareness among pregnant women about obstetric danger signs would lead to an early recognition of complications, thereby causing reduced morbidity and mortality. The study also helped in identifying the influence of various sociodemographic features and obstetric history on the awareness of pregnant women, thereby helping in the elimination of knowledge gaps due to these factors. Additionally, this would contribute to healthcare policies and planning, leading to the development of targeted interventions and public awareness campaigns.

## Figures and Tables

**Figure 1:**
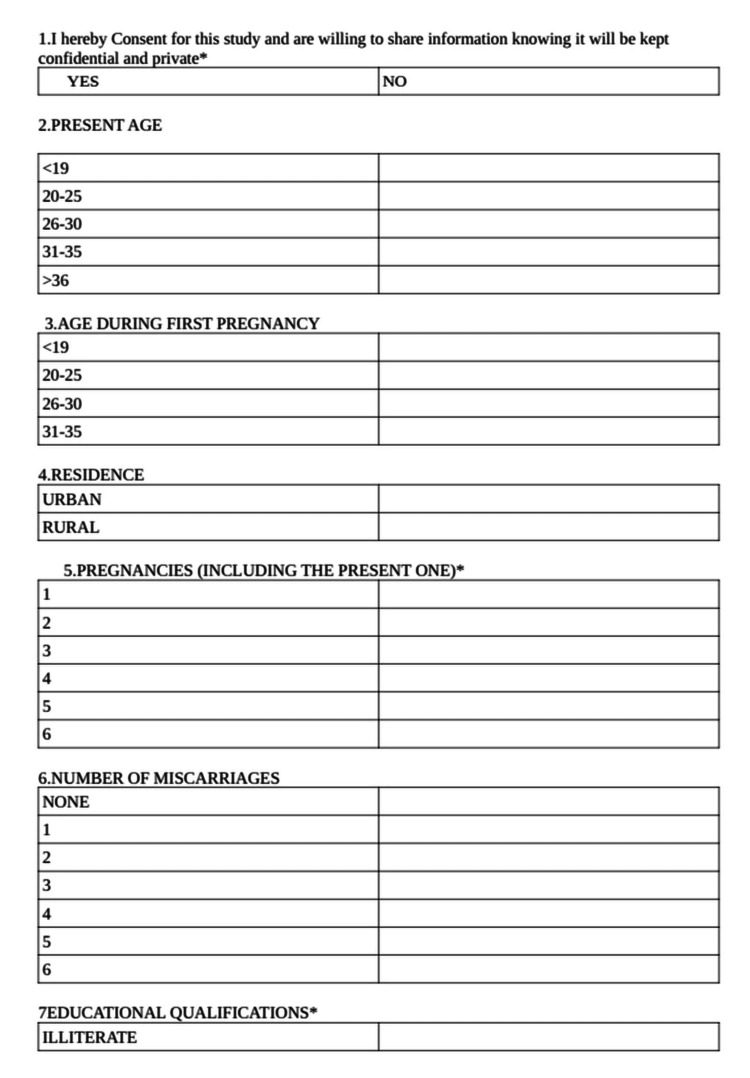
Self-developed questionnaire

**Figure 2:**
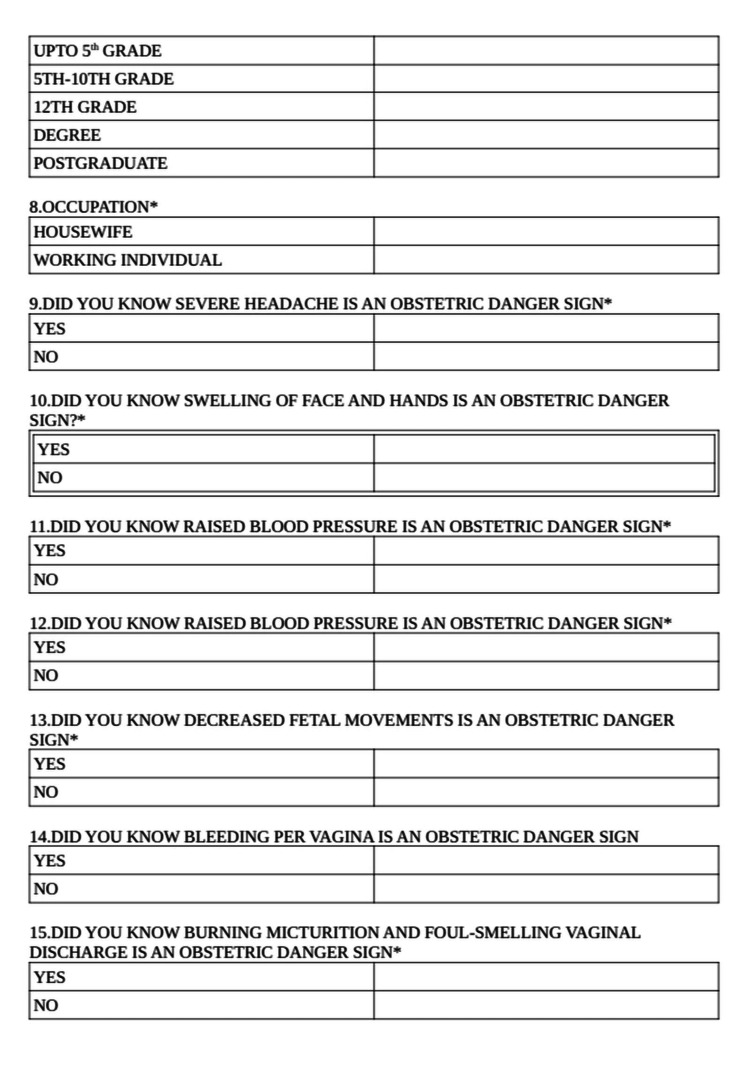
Self-developed questionnaire

**Figure 3:**
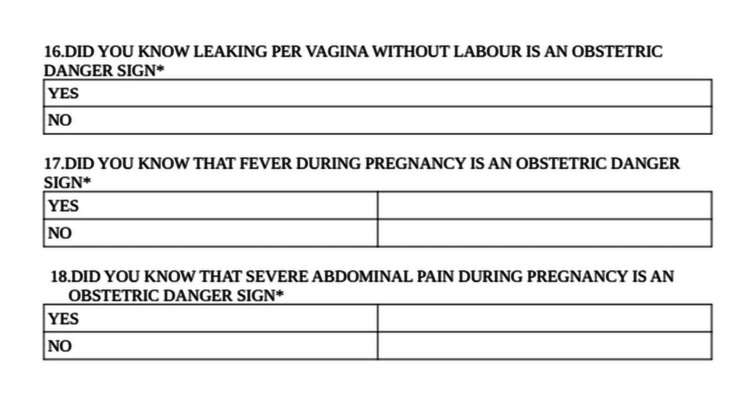
Self-developed questionnaire
